# Remnant cholesterol: a risk stratification aid for coronary artery stenosis severity in patients with type 2 diabetes

**DOI:** 10.3389/fmed.2026.1772328

**Published:** 2026-03-06

**Authors:** Haixiang Gan, Kaijun Gao, Zezhao Li, Yuhong Zhang, Lijuan Li, Yong Li, Hao Guo

**Affiliations:** 1 Department of Cardiology, The First Affiliated Hospital, Kunming Medical University, Kunming, China; 2 Department of Cardiology, Zhaotong Third People’s Hospital, Zhaotong, China

**Keywords:** degree of coronary artery stenosis, lipid, remnant cholesterol, riskstratification, type 2 diabetes

## Abstract

**Objective:**

To investigate the association between remnant cholesterol (RC) levels and the degree of coronary artery stenosis in patients with type 2 diabetes, and to assess its incremental value in risk stratification.

**Methods:**

A retrospective study was conducted on 155 patients with type 2 diabetes diagnosed and treated in the Department of Cardiology at Zhaotong Third People’s Hospital from January 2023 to December 2024. Based on coronary angiography Gensini scores, patients were divided into a mild stenosis group (Gensini < 22 points) and a moderate-to-severe stenosis group (Gensini ≥ 22 points). Clinical characteristics were compared between the two groups. Spearman’s correlation analysis assessed the relationship between RC and Gensini scores. Logistic regression analyzed the association between RC and coronary artery stenosis severity. ROC curves evaluated the diagnostic capability of RC, while NRI and IDI measures assessed its incremental diagnostic value.

**Results:**

The RC in the moderate-to-severe stenosis group was higher than that in the mild stenosis group (*P* < 0.05). RC showed a positive correlation with the Gensini score (*P* < 0.05). Multivariate logistic regression analysis indicated that RC was an independent risk factor for the severity of coronary artery stenosis (OR = 2.849, 95% CI: 1.313–6.181, *P* = 0.008). The AUC for RC in independently identifying coronary artery stenosis was 0.603 (95% CI: 0.514–0.691). Adding RC to the baseline model increased the AUC to 0.790 (95% CI: 0.719–0.860), with statistically significant continuous NRI (0.324, *P* = 0.039) and IDI (0.032, *P* = 0.026).

**Conclusion:**

Levels of RC in patients with type 2 diabetes are independently associated with the degree of coronary artery stenosis. Beyond traditional risk factors, RC demonstrates clear incremental value and can serve as an auxiliary indicator for stratifying the risk of coronary artery stenosis.

## 1 Introduction

Type 2 diabetes is a chronic metabolic disorder characterized by persistent hyperglycemia and dyslipidemia, which significantly increases the risk of cardiovascular disease (1–3). Hyperglycemia promotes oxidative stress through multiple pathways, leading to vascular inflammation, endothelial dysfunction, and cardiovascular injury. It also elevates the risk of coronary thrombosis and directly contributes to myocardial cell damage, thereby participating in the development and progression of coronary heart disease (4, 5).

Extensive research indicates that dyslipidemia, particularly elevated levels of low-density lipoprotein cholesterol (LDL-C), is closely associated with coronary artery plaque formation and vascular stenosis, serving as an independent risk factor for coronary heart disease (6). However, numerous studies have found that even under statin therapy with well-controlled LDL-C levels, some patients still exhibit substantial residual cardiovascular risk (7–9). In recent years, remnant cholesterol (RC) has garnered significant attention as a predictor of cardiovascular events. Multiple studies have confirmed a positive correlation between RC and the risk of cardiovascular events (10–12), and elevated RC levels have been associated with an increased risk of diabetes (13–15). RC and diabetes may interact reciprocally, jointly promoting the occurrence of cardiovascular events. Currently, although RC is associated with cardiovascular events, its direct correlation with the degree of coronary artery stenosis (Gensini score) remains unclear in the high-risk population of type 2 diabetes, with limited reports available. In individuals with type 2 diabetes at high risk for atherosclerotic cardiovascular disease, identifying simple and cost-effective laboratory markers for assessing coronary artery stenosis holds significant clinical value. Therefore, this study investigates the correlation between RC and the degree of coronary artery stenosis in patients with type 2 diabetes, aiming to explore whether RC can serve as an auxiliary assessment indicator for coronary artery stenosis in this population.

## 2 Materials and methods

### 2.1 Study population

A retrospective study was conducted on 155 patients with type 2 diabetes diagnosed and treated in the Department of Cardiology at Zhaotong Third People’s Hospital from January 2023 to December 2024. Based on coronary angiography results, patients were assigned Gensini scores and divided into two groups using the median method: the mild stenosis group (Gensini < 22 points) with 77 cases and the moderate-to-severe stenosis group (Gensini ≥ 22 points) with 78 cases. Inclusion Criteria: (1) Age ≥ 18 years; (2) Meets the 1999 WHO criteria for diabetes diagnosis; (3) Underwent coronary angiography; (4) Complete general clinical data. Exclusion Criteria: (1) Type 1 diabetes, special types of diabetes, etc.; (2) Acute infection within the past 3 months; (3) Severe cardiac diseases such as valvular heart disease, heart failure, or cardiomyopathy; (4) Severe hematologic, immunologic, or hepatic/renal diseases; (5) Patients with malignancies. This study adheres to the Declaration of Helsinki and has received ethical approval from the Medical Ethics Committee of Zhaotong Third People’s Hospital [Approval No.: (2025) Ethics L No. 01].

### 2.2 Research methods

This study collected patients’ general clinical data, including age, gender, history of hypertension, smoking and alcohol consumption history, history of lipid-lowering medication use, duration of diabetes, and body mass index (BMI). Collect relevant laboratory indicators from fasting venous blood drawn the morning after admission, including creatinine (CRE), estimated glomerular filtration rate (eGFR), albumin (ALB), and lipid parameters of the first venous blood during the visit: triglycerides (TG), total cholesterol (TC), high-density lipoprotein cholesterol (HDL-C), and LDL-C. Non-high-density lipoprotein cholesterol (Non-HDL-C) and RC were calculated using the following formulas: Non-HDL-C = TC-HDL-C, RC = TC-HDL-C-LDL-C. Compare the differences in various indicator levels between the mild stenosis group and the moderate-to-severe stenosis group. Conduct univariate and multivariate analyses to investigate the correlation between RC and the degree of coronary artery stenosis.

### 2.3 Gensini score

All patients underwent coronary angiography. Results were independently assessed by three investigators blinded to laboratory findings using the Gensini scoring system to evaluate coronary artery stenosis severity. Scoring was based on objective angiographic images, minimizing subjective influence. Branch scores were calculated as the product of stenosis severity and lesion location coefficients. The total Gensini score represented the sum of scores across all coronary branches (Table 1).

**TABLE 1:** Gensini score.

Severity (%)	Score	Coronary artery branch	Lesion location coefficient
<25%	1	Left main trunk	( × 5)
25–49%	2	Left anterior descending artery	Proximal segment ( × 2.5)
50–74%	4		Middle segment (× 1.5)
75–89%	8		Distal segment (× 1)
90–99%	16	First diagonal branch	(× 1)
100%	32	Second diagonal branch	(× 0.5)
		Left circumflex artery	Proximal segment (× 2.5)
			Middle segment (× 1)
			Distal segment (× 1)
		Blunt margin branch	(× 1)
		Right coronary artery	Proximal segment (× 1)
			Middle segment (× 1)
			Distal segment (× 1)
			Posterior descending artery (× 1)
			Other (× 0.5)
		Posterior left ventricular branch	(× 1)

### 2.4 Statistical analysis

Data statistical analysis was performed using SPSS 27.0 software. Normally distributed information is presented as mean ± standard deviation ($\bar{\text{x}}$ ± *s*), with intergroup comparisons conducted using the independent samples *t*-test. Non-normally distributed information is presented as interquartile range [*M* (Q3–Q1)], with intergroup comparisons performed using the Mann-Whitney *U* test. Categorical variables are presented as numbers (%), with intergroup comparisons conducted using the chi-square test. Spearman correlation analysis was used to evaluate the correlation between each indicator and the Gensini score. Multivariate logistic regression analysis (forward stepwise method, entry criterion α = 0.05, exclusion criterion α = 0.10) was employed to evaluate the association between RC and the severity of coronary artery stenosis. The ROC curve was used to assess the discriminatory ability of RC in distinguishing the degree of coronary artery stenosis in patients with type 2 diabetes.

To assess the incremental value of RC, five logistic regression models were constructed: Model 1 (baseline model) included age, sex, hypertension, smoking, alcohol consumption, lipid-lowering medication, diabetes duration, CRE, eGFR, and LDL-C; Model 2 added RC to Model 1; Models 3–5 evaluated the baseline efficacy of RC combined with other biomarkers, incorporating only RC+CRE, RC+LDL-C, and RC+CRE+LDL-C, respectively, without adjusting for other covariates. Area under the curve (AUC) and 95% confidence intervals (CI) were calculated for each model using ROC curves, with AUC differences compared via the DeLong test. The discriminative improvement of Model 2 over Model 1 was assessed using the net reclassification index (NRI) and integrated discrimination improvement (IDI). DeLong tests, NRI, and IDI were performed using R software (version 4.4.1). The calibration of the models was assessed using the Hosmer-Lemeshow goodness-of-fit test, with *P* > 0.05 indicating adequate calibration. *P* < 0.05 was considered statistically significant.

This study estimated the sample size based on the AUC values of RC for predicting the severity of coronary artery lesions in published literature (16). Using MedCalc software, with an expected AUC of 0.63, a null hypothesis AUC of 0.5, α = 0.05 (two-tailed), a power of 80%, and a 1:1 sample size ratio between groups, the exact method yielded a minimum total sample size of 152 cases (76 in the positive group and 76 in the negative group). This study actually enrolled 155 subjects, meeting the sample size requirement.

## 3 Results

### 3.1 Baseline characteristics

Table 2 shows that the BMI was higher in the mild stenosis group than in the moderate-to-severe stenosis group (*P* < 0.05). The duration of diabetes, smoking prevalence, and alcohol consumption prevalence were higher in the moderate-to-severe stenosis group than in the mild stenosis group (*P* < 0.01), with smokers and drinkers being exclusively male. Comparisons of age distribution, gender composition, hypertension prevalence, and lipid-lowering medication use between the two groups showed no statistically significant differences (*P* > 0.05). Regarding laboratory indicators, the moderate-to-severe stenosis group exhibited higher levels of CRE, TC, LDL-C, Non-HDL-C, and RC compared to the mild stenosis group (*P* < 0.05), while eGFR was lower in the moderate-to-severe stenosis group (*P* < 0.01). Differences in ALB, TG, and HDL-C levels between the two groups were not statistically significant (*P* > 0.05).

**TABLE 2:** Baseline feature comparison [($\bar{\text{X}}$ ± *s*),*M* (Q3–Q1)].

Characteristics	Mild group (*n* = 77)	Moderate-to-Severe Group (*n* = 78)	*t/x*^2^/-*z*-value	*P*-value
Age (years)	63.53 ± 10.66	63.62 ± 9.67	−0.051	0.960
Gender [n (%)]			2.888	0.089
Male	30 (38.96)	41 (52.56)		
Female	47 (61.04)	37 (47.44)		
Hypertension [n(%)]	58 (75.32)	56 (71.79)	0.248	0.618
Smoking [n(%)]	16 (20.78)	35 (44.87)	10.188	0.001
Alcohol consumption [n(%)]	10 (12.99)	30 (38.46)	13.133	<0.001
Lipid-lowering drugs [n(%)]	71 (92.21)	74 (94.87)	0.456	0.500
Duration of diabetes (years)	4.00 (2.00, 9.50)	8.71 (3.00, 12.00)	2.745	0.006
BMI (kg/m^2^)	26.10 ± 3.46	24.51 (23.05, 26.54)	−2.451	0.014
CRE (μmol/L)	60.48 ± 14.90	66.80 (60.05, 84.00)	4.030	<0.001
eGFR (mL/min/1.73 m^2^)	95.21 ± 13.51	90.18 (80.07, 96.45)	−3.239	0.001
ALB (g/L)	41.74 ± 3.20	42.19 ± 4.47	−0.727	0.469
TC (mmol/L)	4.68 (3.92, 5.12)	4.70 (4.27, 5.40)	2.554	0.011
TG (mmol/L)	1.90 (1.23, 2.23)	2.12 (1.26, 2.47)	1.311	0.190
HDL-C (mmol/L)	1.09 (0.94, 1.20)	1.09 (0.94, 1.19)	−0.318	0.751
LDL-C (mmol/L)	2.84 (2.17, 3.04)	2.84 (2.68, 3.29)	2.064	0.039
Non-HDL-C (mmol/L)	3.63 (2.79, 4.08)	3.66 (3.22, 4.24)	2.069	0.039
RC (mmol/L)	0.66 (0.31, 0.72)	0.69 (0.48, 0.95)	2.218	0.027

Normally distributed information is presented as mean ± standard deviation ($\bar{\text{x}}$ ± *s*). Non-normally distributed information is presented as interquartile range [*M* (Q3–Q1)]. Categorical variables are presented as n(%). BMI, body mass index; CRE, creatinine; eGFR, estimated glomerular filtration rate; ALB, albumin; TC, total cholesterol; TG, triglycerides; HDL-C, high-density lipoprotein cholesterol; LDL-C, low-density lipoprotein cholesterol; Non-HDL-C, non-high-density lipoprotein cholesterol; RC, remnant cholesterol. *P* < 0.05 was considered statistically significant.

### 3.2 Spearman’s correlation analysis

As shown in Table 3, diabetes duration, CRE, TC, TG, LDL-C, Non-HDL-C, and RC were positively correlated with the Gensini score (*P* < 0.05), while eGFR was negatively correlated with the Gensini score (*P* < 0.01). ALB and HDL-C showed no significant correlation with the Gensini score (*P* > 0.05).

**TABLE 3:** Correlation analysis between Gensini score and various parameters in type 2 diabetes patients.

Indicators	*r-*value	*P-*value
Duration of diabetes	0.217	0.007
CRE	0.305	< 0.001
eGFR	−0.215	0.007
ALB	0.124	0.125
TC	0.227	0.004
TG	0.173	0.031
HDL-C	−0.062	0.442
LDL-C	0.234	0.003
Non-HDL-C	0.201	0.012
RC	0.179	0.026

CRE, creatinine; eGFR, estimated glomerular filtration rate; ALB, albumin; TC, total cholesterol; TG, triglycerides; HDL-C, high-density lipoprotein cholesterol; LDL-C, low-density lipoprotein cholesterol; Non-HDL-C, non-high-density lipoprotein cholesterol; RC, remnant cholesterol. *P* < 0.05 was considered statistically significant.

### 3.3 Multivariate logistic regression analysis

Forward stepwise regression was employed for multivariate logistic regression analysis. The variable selection process proceeded as follows: Based on clinical significance and univariate analysis results, age, sex, hypertension, smoking, alcohol consumption, lipid-lowering medication, diabetes duration, CRE, eGFR, TC, TG, LDL-C, Non-HDL-C, and RC were included as candidate independent variables. Stepwise regression employed likelihood ratio tests, with variables entering the model at *P* < 0.05 and excluded at *P* > 0.10. Although BMI showed statistical significance in univariate analysis (*P* < 0.05), its distribution was higher in the mild stenosis group than in the moderate-to-severe stenosis group, contrary to clinical expectations. To avoid reverse confounding, it was excluded from the model. ALB and HDL-C were also excluded due to lack of statistical significance in univariate analysis (*P* > 0.05). The final results are shown in Table 4. RC and CRE were independent risk factors for the degree of coronary artery stenosis in patients with type 2 diabetes (RC: OR = 2.849, 95% CI: 1.313–6.181; CRE: OR = 1.037, 95% CI: 1.012–1.062).

**TABLE 4:** Analysis of independent risk factors for coronary artery stenosis in patients with type 2 diabetes.

Indicators	β	Standard Error	Wald χ^2^	Odds Ratio	95% CI	*P-*value
RC	1.047	0.395	7.018	2.849	1.313–6.181	0.008
CRE	0.036	0.012	8.675	1.037	1.012–1.062	0.003

RC, remnant cholesterol; CRE, creatinine. *P* < 0.05 was considered statistically significant.

### 3.4 ROC curve analysis

**FIGURE 1:**
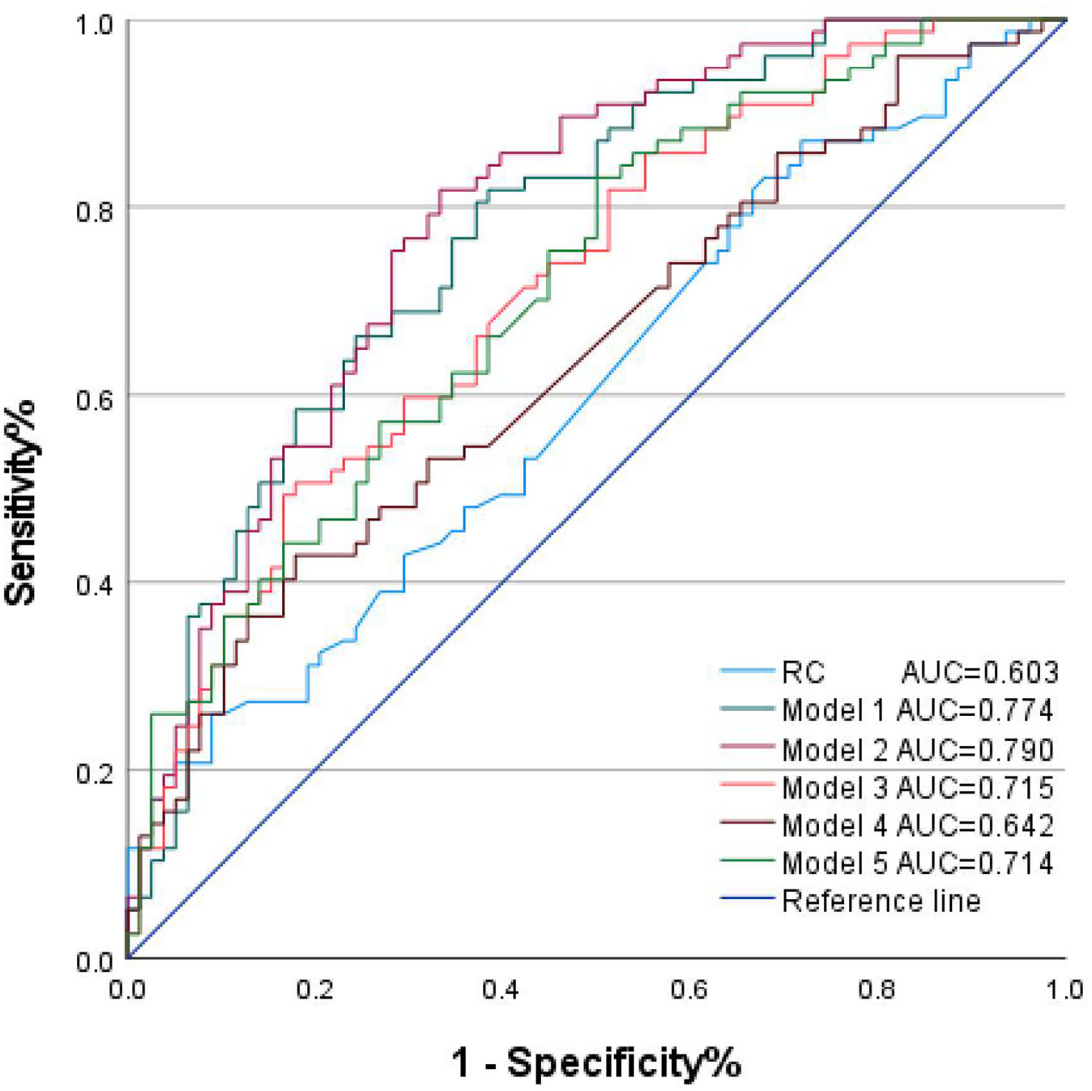
ROC curve for assessing the efficacy of determining the degree of coronary artery stenosis in type 2 diabetes patients. Model 1 (Baseline Model): Age, Gender, Hypertension, Smoking, Alcohol Consumption, Lipid-Lowering Medication, Diabetes Duration, CRE, eGFR, LDL-C; Model 2 (Incremental Model): Model 1 + RC; Model 3: RC + CRE; Model 4: RC + LDL-C; Model 5: RC + CRE + LDL-C. Models 3–5 were not adjusted for other covariates. RC, remnant cholesterol; CRE, creatinine.; eGFR, estimated glomerular filtration rate; LDL-C, low-density lipoprotein cholesterol.

ROC curves were used to evaluate the discriminatory performance of each indicator and model for assessing the degree of coronary artery stenosis in patients with type 2 diabetes. Results are shown in Table 5 and Figure 1. The AUC for RC alone was 0.603 (95% CI: 0.514–0.691), while Model 1 (baseline model) achieved an AUC of 0.774 (95% CI: 0.701–0.847). Adding RC to Model 1 improved Model 2’s AUC to 0.790 (95% CI: 0.719–0.860). Among biomarker-only models, Model 3 (RC+CRE) and Model 5 (RC+CRE+LDL-C) yielded AUC values of 0.715 (95% CI: 0.635–0.795) and 0.714 (95% CI: 0.635–0.794), respectively, both higher than Model 4 (RC+LDL-C) at 0.642 (95% CI: 0.556–0.728). Detailed parameters for the predictive performance of each model (Yardene index, cutoff value, sensitivity, and specificity) are presented in Table 5.

**TABLE 5:** Discriminatory performance of RC and various models for assessing the severity of coronary artery stenosis in type 2 diabetes patients.

Indicators	Yorden Index	Cut-off value	Sensitivity (%)	Specificity (%)	AUC (95% CI)	Standard Error	*P*-value
RC	0.170	0.315	0.260	0.910	0.603 (0.514–0.691)	0.045	0.027
Model 1	0.433	0.546	0.805	0.628	0.774 (0.701–0.847)	0.037	0.000
Model 2	0.485	0.524	0.818	0.667	0.790 (0.719–0.860)	0.036	0.000
Model 3	0.327	0.409	0.506	0.821	0.715 (0.635–0.795)	0.041	0.000
Model 4	0.250	0.446	0.429	0.821	0.642 (0.556–0.728)	0.044	0.002
Model 5	0.302	0.458	0.571	0.731	0.714 (0.635–0.794)	0.041	0.000

Model 1 (Baseline Model): Age, Gender, Hypertension, Smoking, Alcohol Consumption, Lipid-Lowering Medication, Diabetes Duration, CRE, eGFR, LDL-C; Model 2 (Incremental Model): Model 1 + RC; Model 3: RC + CRE; Model 4: RC + LDL-C; Model 5: RC + CRE + LDL-C. Models 3–5 were not adjusted for other covariates. *P* < 0.05 was considered statistically significant. RC, remnant cholesterol; CRE, creatinine.; eGFR, estimated glomerular filtration rate; LDL-C, low-density lipoprotein cholesterol.

The incremental value assessment is shown in Table 6. Compared with Model 1 (baseline model), Model 2 incorporating RC demonstrated certain incremental value. The continuous NRI for Model 2 was 0.324 (*P* = 0.039) and the IDI was 0.032 (*P* = 0.026), both differences being statistically significant; however, the categorical NRI was 0.104 (*P* = 0.175), failing to reach statistical significance. DeLong test results (Table 7) indicate that compared to RC alone, Model 3 (RC+CRE) and Model 5 (RC+CRE+LDL-C) both showed significantly improved AUC (ΔAUC = 0.112, *P* = 0.008; ΔAUC = 0.112, *P* = 0.009), while Model 4 (RC+LDL-C) showed no significant improvement (ΔAUC = 0.039, *P* = 0.210). Further comparison revealed that both Model 3 and Model 5 had higher AUC values than Model 4, though the differences did not reach statistical significance (*P* = 0.116; *P* = 0.077).

**TABLE 6:** Incremental value of model 2 compared to model 1.

Indicators	Valuation	95% CI	*P-*value
Categorical NRI	0.104	−0.046–0.254	0.175
Continuous NRI	0.324	0.017–0.632	0.039
IDI	0.032	0.004–0.060	0.026

NRI, net reclassification index; IDI, integrated discrimination improvement.

**TABLE 7:** DeLong test compares the AUC differences among different models.

Comparison group	AUC	ΔAUC (95% CI)	*z*-value	*P*-value
Model 3 vs. RC Standalone	0.715 vs. 0.603	0.112 (0.029–0.195)	2.638	0.008
Model 4 vs. RC Standalone	0.642 vs. 0.603	0.039 (−0.022–0.100)	1.254	0.210
Model 5 vs. RC Standalone	0.714 vs. 0.603	0.112 (0.028–0.195)	2.615	0.009
Model 3 vs. Model 4	0.715 vs. 0.642	0.073 (−0.018–0.164)	1.573	0.116
Model 5 vs. Model 4	0.714 vs. 0.642	0.072 (−0.008–0.153)	1.768	0.077

Model 3: RC + CRE; Model 4: RC + LDL-C; Model 5: RC + CRE + LDL-C. Models 3–5 were not adjusted or other covariates. RC, remnant cholesterol; CRE, creatinine.; LDL-C, low-density lipoprotein cholesterol.

## 4 Discussion

The primary findings of this study are as follows: (1) Among patients with type 2 diabetes, the RC levels were higher in the group with moderate-to-severe coronary artery stenosis compared to the group with mild stenosis; (2) RC levels were positively correlated with Gensini scores and constituted an independent risk factor for the severity of coronary artery stenosis; (3) ROC curve analysis indicated that RC alone had limited discriminatory ability for coronary artery stenosis severity, but when combined with other indicators, the model’s discriminatory efficacy significantly improved. Further analysis demonstrated that RC provided significant incremental value to the baseline model, with both continuous NRI and IDI showing statistical significance.

RC refers to cholesterol content other than HDL-C and LDL-C, specifically the cholesterol carried by cholesterol-rich lipoprotein particles remaining after the metabolism of triglyceride-rich lipoproteins (TRLs). This includes residual particles such as chylomicrons, very low-density lipoprotein cholesterol, and medium-density lipoprotein cholesterol (17). Numerous studies demonstrate that TRLs and RC constitute significant components of residual cardiovascular risk (18–21). Some patients exhibit substantial residual cardiovascular risk despite markedly reduced LDL-C levels. Therefore, RC measurement may aid in identifying potential cardiovascular disease not fully reflected by LDL-C. Research even indicates that RC outperforms LDL-C in predicting arterial stiffness progression (22). Multiple studies have demonstrated that RC levels correlate with major adverse cardiovascular events in both primary and secondary prevention of atherosclerotic cardiovascular disease (23–26). Furthermore, research indicates that elevated RC levels constitute an independent risk factor for atherosclerotic cardiovascular disease, independent of traditional risk factors such as LDL-C levels. This suggests RC may hold significant value in primary prevention of cardiovascular disease (27–29). Additional research indicates that elevated RC levels may serve as a predictor of coronary outcomes in patients with type 2 diabetes and could increase mortality risk (30, 31). Furthermore, studies have found that prediabetic individuals with higher RC levels exhibit a greater propensity to develop diabetes compared to those with normal blood glucose levels (32). This study focused on the high-risk population of type 2 diabetes patients, linking RC with the degree of coronary artery stenosis. The results align with previous research, suggesting that RC is not only associated with cardiovascular events but also closely related to the severity of vascular structural lesions. By advancing the endpoint from “events” to “structural stenosis,” this study provides a basis for more in-depth mechanistic research into the atherogenic effects of RC.

Multiple studies have confirmed the association between RC and atherosclerotic cardiovascular disease, but the mechanism by which RC induces atherosclerosis remains incompletely understood. Existing research suggests it may be closely linked to a vicious cycle involving oxidative stress and inflammatory responses (16, 33). Atherosclerosis initiates with vascular endothelial injury and associated inflammatory reactions. Circulating RC can invade and become trapped within arterial walls, directly damaging the vascular endothelium. Compared to LDL-C, RC particles do not require oxidative modification and exhibit stronger binding affinity to macrophages. Macrophages can directly recognize and phagocytose RC particles, forming foam cells that secrete large amounts of inflammatory mediators, leading to vascular endothelial damage and inflammatory responses. Additionally, RC particles are larger in size and carry more cholesterol, making them more likely to induce atherosclerotic plaque formation. RC induces oxidative stress, activating transcription factors that regulate inflammatory mediators and exacerbate inflammatory responses, thereby promoting plaque growth. This inflammation further stimulates local reactive oxygen species production, creating a vicious cycle that destabilizes plaques and triggers adverse cardiovascular events. Additionally, RC induces the expression of thrombogenic molecules in the vascular endothelium while activating coagulation and inhibiting fibrinolysis, further accelerating atherosclerosis progression. In summary, elevated RC levels contribute to and accelerate atherosclerosis progression through oxidative stress, inflammation, and thrombosis. Metabolic dysregulation in type 2 diabetes patients synergizes with RC elevation, increasing the risk of adverse cardiovascular events.

In this study, RC levels in patients with type 2 diabetes were significantly correlated with the degree of coronary artery stenosis and constituted an independent risk factor. However, RC demonstrated limited discriminatory ability in identifying patients with moderate-to-severe stenosis alone (AUC = 0.603). This finding differs from some previous studies, potentially due to the majority of patients already taking lipid-lowering medications, which may have weakened RC’s discriminatory capacity, and the cross-sectional design’s inability to reflect long-term exposure effects. Nevertheless, this outcome reflects the complexity and multifactorial nature of coronary artery disease progression. This result is close to the AUC (0.626) reported by Chen Xiao Yun et al. (16), and both studies showed specificity > 90%, indicating that high specificity is a stable characteristic of RC. Although RC exhibits low sensitivity (26.00%), its high specificity of 91.00% suggests that while its ability to identify all patients with moderate-to-severe stenosis is limited, elevated RC levels hold significant value in identifying those with substantial stenosis. This makes RC a valuable “risk-enhancing” warning indicator with complementary clinical utility.

Adding RC to the baseline model improved the model’s discriminatory performance, with AUC increasing from 0.774 to 0.790. The continuous NRI value of 0.324 indicates that Model 2 correctly reclassified 32.4% more patients than Model 1, demonstrating a significant enhancement in risk stratification capability. The IDI value of 0.032 reflects a small but statistically significant improvement in average sensitivity without compromising specificity. This suggests that RC provides clear incremental value, consistent with recent research on the role of remnant lipoproteins in residual cardiovascular risk (19). In simplified biomarker-only models, Models 3 (RC+CRE) and 5 (RC+CRE+LDL-C) exhibited higher AUC values (0.715, 0.714) than Model 4 (RC+LDL-C, 0.642), though DeLong tests failed to reach statistical significance (*P* = 0.116; *P* = 0.077). This trend suggests a potential synergistic effect between RC and CRE. As a renal function marker, CRE may capture additional pathophysiological mechanisms associated with diabetic nephropathy and atherosclerosis, thereby enhancing predictive capability when combined with RC. This simple composite model holds potential as a risk screening tool in resource-limited settings, though validation in larger-scale studies is warranted.

The findings of this study provide important implications for clinical lipid management: In patients with type 2 diabetes who have achieved LDL-C targets but exhibit elevated RC, this increased RC may indicate the need to focus on residual cardiovascular risk, thereby warranting intensified lipid-lowering therapy or more proactive imaging assessment (34, 35). However, RC has limited discriminatory ability on its own and is not recommended as an independent screening tool. It can, however, serve as a valuable supplement to traditional risk assessment models.

### 4.1 Limitations and research prospects

This study has the following limitations: First, the single-center cross-sectional design precludes inference of causality. Second, the relatively limited sample size may affect statistical power. Third, most patients were already taking lipid-lowering medications, which may have reduced lipid levels and thereby weakened the independent discriminatory ability of RC. However, there was no significant difference in medication use between the two groups, and RC remained an independent risk factor after adjusting for medication status, suggesting its impact on the study conclusions was limited. Fourth, RC was calculated indirectly via a formula rather than measured directly. In patients with significantly elevated TG, calculation errors in LDL-C may have affected RC accuracy. However, TG levels were generally well-controlled in this population, suggesting limited impact of calculation errors on conclusions. Fifth, although multiple related lipid markers were included in this study, potentially introducing multicollinearity, the RC effect remained stable after stepwise regression selection (95% CI: 1.313–6.181, excluding 1), suggesting that multicollinearity had limited impact on the primary conclusions. Future prospective multicenter studies with expanded sample sizes and standardized lipid management at baseline could more accurately assess the relationship between RC changes and coronary lesion progression. Interventional studies exploring RC’s clinical value in guiding lipid-lowering therapy are also warranted. Integrating additional indicators could facilitate the development of more precise cardiovascular risk assessment models for type 2 diabetes patients.

## 5 Conclusion

In summary, this study found that RC levels in patients with type 2 diabetes are independently associated with the degree of coronary artery stenosis. Although RC has limited independent discriminatory ability, its simplicity, low cost, and high specificity, coupled with its clear incremental value beyond traditional risk factors, suggest potential as a complementary indicator to conventional risk assessment. This offers new insights for individualized cardiovascular risk prevention and treatment in patients with type 2 diabetes.

## Data Availability

The raw data supporting the conclusions of this article will be made available by the authors, without undue reservation.
